# Whole-Genome Sequences of Two Salmonella enterica Serovar Dublin Strains That Harbor the *viaA*, *viaB*, and *ompB* Loci of the Vi Antigen

**DOI:** 10.1128/MRA.00028-19

**Published:** 2019-04-04

**Authors:** Manal Mohammed, Marie-Leone Vignaud, Sabrina Cadel-Six

**Affiliations:** a School of Life Sciences, University of Westminster, London, United Kingdom; b Université Paris-Est, Marne-la-Vallée, France; c Salmonella and Listeria Unit, ANSES, Laboratory for Food Safety, Maisons-Alfort, France; University of Southern California

## Abstract

Here, we report the genome sequences of two Salmonella enterica serovar Dublin strains, 03EB8736SAL and 03EB8994SAL, isolated from raw-milk cheese and milk filtrate, respectively. Analysis of the draft genomes of the two isolates reveals the presence of the *viaA*, *viaB,* and *ompB* loci of the Vi capsular polysaccharide antigen (Vi antigen).

## ANNOUNCEMENT

Salmonella enterica serovar Dublin is adapted to cattle and can be transmitted to humans via the consumption of contaminated raw milk and raw-milk cheeses (1, 2). Human infection by S. Dublin is primarily characterized by self-limiting gastrointestinal illness; however, a high proportion of S. Dublin cases are associated with systemic illness (3, 4). Fatal human outbreaks of S. Dublin were recently reported in France due to the consumption of raw-milk cheese (5).

Vi capsular polysaccharide antigen (Vi antigen) is commonly found in strains of human-adapted typhoidal Salmonella enterica serovars Typhi and Paratyphi (6). It contributes to bacterial virulence and pathogenesis (7, 8). The presence of Vi antigen within nontyphoidal S. Dublin strains might contribute to bacterial virulence and invasiveness. Although the Vi antigen was detected in some strains of S. Dublin (3, 9), it is not very common (4, 10). Expression of the Vi antigen is controlled by 3 chromosomal loci, *viaA*, *viaB,* and *ompB*. Both *viaA* and *viaB* loci are harbored by Salmonella pathogenicity island 7 (SPI-7) (11). The *viaA* locus contains genes that are present not only in Vi-expressing strains of Salmonella but also in Escherichia coli and Citrobacter species (12). The *viaB* locus is composed of 11 genes, including 5 genes for Vi biosynthesis (*tivA, tivB, tivC, tivD,* and *tivE*), 5 genes for Vi antigen export (*vexA, vexB, vexC, vexD,* and *vexE*), and open reading frame 11 (ORF11). The *ompB* locus contains two regulatory systems (*rscB-rscC* and *ompR-envz*), and it controls the regulation of Vi polysaccharide synthesis (8). Here, we report the draft genome sequences of two S. Dublin strains, 03EB8736SAL and 03EB8994SAL, isolated from raw-milk cheese and milk filtrate, respectively, that harbor the three chromosomal loci of the Vi antigen.

The two strains, 03EB8736SAL and 03EB8994SAL, were identified as belonging to Salmonella Dublin according to the White-Kauffmann-Le Minor scheme (https://www.pasteur.fr/sites/default/files/veng_0.pdf). Genomic DNA was extracted using the QIAamp DNA minikit (Qiagen, UK). The quality of DNA was checked using gel electrophoresis, and DNA quantity was determined using the Qubit quantification platform (Invitrogen, USA). Genomic DNA libraries were prepared using the Nextera XT library preparation kit (Illumina, San Diego, CA, USA), following the manufacturer’s protocol. Whole-genome sequencing (WGS) of multiplexed libraries was carried out on the Illumina HiSeq platform using a 250-bp paired-end protocol. The total number of reads for each S. Dublin isolate is provided in Table 1.

**TABLE 1:** Assembly data for Salmonella Dublin strains sequenced in this study

Parameter	Value or result for strain:	
	03EB8736SAL	03EB8994SAL
No. of raw reads	1,092,502	480,292
Mean coverage (×)	91.95	38.60
Assembly parameters		
No. of contigs	30	32
Size of draft genome (bp)	4,993,782	4,994,000
GC content (%)	52.18	52.18
*N*_50_ value	432,097	458,013
Vi antigen and plasmids, accession no. (% identity)		
*viaA*, *viaB,* and *ompB* of Vi antigen, AL513382	Present (99)	Present (99)
pSE81-1705, NZ_CP018654	Present (99.83)	Present (99.83)
pOU1113, NC_007208	Present (99.61)	Present (99.61)
pSPUV, NC_019112	Present (99.80)	Present (99.80)
pCFSAN000725_01, NZ_CP012348	Present (99.74)	Present (99.74)
pQJDsal2, NZ_CP022965	Present (99.38)	Present (99.38)
pSG, HG970001	Present (99.72)	Present (99.72)
Plasmid 3, NZ_LN868945	Present (98.72)	Present (98.72)

**FIG 1:**
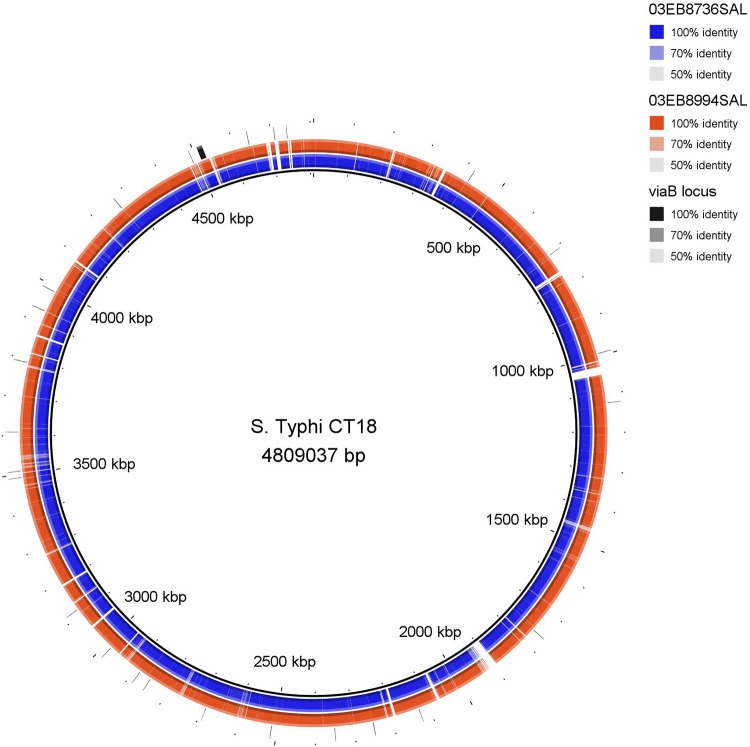
Complete genome alignment of the two *Salmonella* Dublin strains, 03EB8736SAL and 03EB8994SAL, generated using BRIG (15). The three chromosomal loci of the Vi antigen, including the *viaB* locus, are present in the two *Salmonella* Dublin strains as well as in the reference strain of *Salmonella* Typhi strain CT18 (GenBank accession number AL513382).

The quality of Illumina sequencing data was evaluated using FastQC toolkit version 0.11.7 (http://www.bioinformatics.babraham.ac.uk/projects/fastqc/). Adapter sequences were removed using ea-utils package version 1.04.807 (https://expressionanalysis.github.io/ea-utils/). Sequencing data were *de novo* assembled using SPAdes version 3.11 (13) (Fig. 1). SPAdes was run using different assembly parameters looking for best assembly with the highest *N*_50_ value and largest contig size. The quality of multiple assemblies were compared using Quality Assessment Tool for Genome Assemblies (QUAST) (http://quast.bioinf.spbau.ru/). The assembly data (size of draft genome, number of contigs, GC content, and *N*_50_ value) for each S. Dublin isolate are provided in Table 1. The virulence genes involved with the *viaB* locus of the Vi antigen were determined using BLASTn version 2.2.25 (14) with above 90% similarity. BLAST Ring Image Generator (BRIG) version 0.95-dev.0004 (15) was used to illustrate the presence of Vi chromosomal loci within the draft genomes of the two S. Dublin strains, and Salmonella enterica serovar Typhi strain CT18 harboring Vi antigen (GenBank accession number AL513382) was used as a reference. Moreover, analysis of harbored plasmid/s within the draft genome of the two S. Dublin isolates, 03EB8736SAL and 03EB8994SAL, was performed using PLSDB with search strategy Mash screen, and the default values were a maximum *P* value of 0.1 and minimum identity of 0.99 (https://ccb-microbe.cs.uni-saarland.de/plsdb/) (16). Interestingly, seven plasmids that were heterogeneous in size (33 to 147 kb) were detected within the draft genomes, as shown in Table 1. Plasmids can harbor virulence genes required to trigger systemic illness (17).

The presence of Vi chromosomal loci within nontyphoidal S. Dublin strains might play an important role in the ability of bacteria to cause invasive illness in humans. Further studies will be carried out to evaluate the expression of Vi antigen and determine the exact role of Vi antigen in S. Dublin virulence and pathogenesis.

### Data availability.

The raw sequence reads and draft assemblies of the two S. Dublin isolates, 03EB8736SAL and 03EB8994SAL, have been deposited in the European Nucleotide Archive (ENA) under project number PRJEB30372. The raw sequence reads are available under accession numbers ERS3015385 and ERS3015387, and the draft genome sequences are available under accession numbers ERS3015643 and ERS3015644, respectively.
